# Patient Characteristics, Diagnostic Delays, Treatment Patterns, Treatment Outcomes, Comorbidities, and Treatment Costs of Acromegaly in China: A Nationwide Study

**DOI:** 10.3389/fendo.2020.610519

**Published:** 2020-12-01

**Authors:** Xiaopeng Guo, Kailu Wang, Siyue Yu, Lu Gao, Zihao Wang, Huijuan Zhu, Bing Xing, Shuyang Zhang, Dong Dong

**Affiliations:** ^1^Department of Neurosurgery, Peking Union Medical College Hospital, Chinese Academy of Medical Sciences and Peking Union Medical College, Beijing, China; ^2^Key Laboratory of Endocrinology of Ministry of Health, Peking Union Medical College Hospital, Chinese Academy of Medical Sciences and Peking Union Medical College, Beijing, China; ^3^China Pituitary Disease Registry Center, Beijing, China; ^4^China Pituitary Adenoma Specialist Council, Beijing, China; ^5^JC School of Public Health and Primary Care, Faculty of Medicine, The Chinese University of Hong Kong, Hong Kong, China; ^6^Department of Endocrinology, Peking Union Medical College Hospital, Chinese Academy of Medical Sciences and Peking Union Medical College, Beijing, China; ^7^China Alliance of Rare Diseases, Beijing, China; ^8^Department of Cardiology, Peking Union Medical College Hospital, Chinese Academy of Medical Sciences and Peking Union Medical College, Beijing, China; ^9^Shenzhen Research Institute of Chinese University of Hong Kong, Shenzhen, China

**Keywords:** acromegaly, diagnostic delay, treatment patterns, comorbidity, economic burden, China, endocrine remission

## Abstract

**Purpose:**

Acromegaly is a rare, intractable endocrine disease. We aimed to describe the patient characteristics, diagnostic delays, treatment patterns, treatment outcomes, comorbidities and treatment costs of acromegaly in China.

**Methods:**

This is a nationwide cross-sectional study. Patients diagnosed with and treated for acromegaly between 1996 and 2019 across China were surveyed via the Chinese Association of Patients with Acromegaly platform.

**Results:**

In total, 473 patients (58.8% females, mean age at diagnosis: 39.4±9.5 years) were included. The median disease duration was 3 years. The most common symptoms were extremity enlargement (91.8%) and facial changes (90.1%). Overall, 63.0% of patients experienced diagnostic delays within healthcare systems; 63.8% of the delays were <1 year. The most common first-line therapy was surgery with a transsphenoidal (76.1%) or transcranial approach (3.2%). Somatostatin analogues or dopamine agonists were administered in 20.5% of the patients as first-line therapies and in 41.7% as adjuvant therapies. Radiotherapy was performed in 32.1% of patients, 99.3% of whom received radiotherapy as an adjuvant therapy. After a median 5-year follow-up, 46.2% achieved biochemical control. Comorbidities were reported in 88.2% of the patients at follow-up; memory deterioration and thyroid nodules were the most common. Controlled patients had greater improvements in symptoms and comorbidities during follow-up than uncontrolled patients. The annual per-capita cost-of-treatment was $11013 in 2018, with medical treatments being the largest contributor (67%). Medical insurance covered 47.2% of all treatment costs.

**Conclusion:**

This study provides the first comprehensive description of real-world acromegaly data in China, serving as a basis for future population-based studies.

## Introduction

Acromegaly, caused by a growth hormone (GH)-secreting pituitary adenoma in most cases, is a rare, slow progressing endocrine disease (1). Worldwide, acromegaly has a prevalence of 28–137 per million and an incidence ranging from 2 to 11 cases per million people per year (2). GH over-secretion leads to increased serum insulin-like growth factor 1 (IGF-1), and both hormones result in a series of acromegaly-associated symptoms, i.e., facial appearance changes; overgrowth of hands and feet; headache; visual field defects; and comorbidities affecting the cardiovascular system, respiratory system, musculoskeletal organs, endocrine/metabolic system, etc (3–7). Since the above clinical features of acromegaly develop insidiously, the diagnosis of this condition is therefore often delayed, and prolonged diagnostic delays are associated with reduced quality of life (QoL) and elevated morbidity and mortality in patients with acromegaly (7–9). The main therapeutic goals include normalization of the circulating levels of GH and IGF-1, removal of pituitary tumors without damaging the adjacent structures, amelioration of symptoms and comorbidities, and restoration of normal life expectancy and QoL (3, 4, 10). Treatment options for acromegaly include surgical resection of pituitary adenomas, medical treatment to control hormone secretion and tumor growth, and radiotherapy, with transsphenoidal surgery being the first-line treatment for most patients (4, 7). Medical treatment also has an important role in acromegaly management, either as the first-line therapy for patients with high surgical risks and those who refuse surgery or as second-line treatment (4, 10, 11). Radiotherapy acts as a third-line treatment after unsuccessful surgery and medical treatment (12). Routine post-treatment follow-up is essential for acromegaly. In patients who achieve biochemical control, life expectancy can be restored to that of the general population (4, 13). However, tumor recurrence and high hormone concentrations occur in almost half of patients after treatment, leading to persistent symptoms and comorbidities and thus deteriorating patient prognosis and influencing the overall well-being of these patients (4, 7). Apart from the negative health-related effects, acromegaly also leads to a considerable economic burden for patients themselves, their families, the healthcare system and society as a whole (14, 15).

Studies based on national registries or large-scale nationwide surveys have provided an in-depth understanding of the epidemiology, clinical features, diagnostic procedures, and treatment patterns and outcomes of patients with acromegaly (16–24). Region-specific data greatly help a country or region formulate medical policies related to the diagnosis and treatment of acromegaly, which in turn improve patient prognosis (23). However, no nationwide or large-scale study on the real-world data of acromegaly in China has been conducted until now. China has over 1.4 billion people and a large number of patients with acromegaly, but unfortunately, a complete and functioning national acromegaly registry has not been established as of now. The Chinese Association of Patients with Acromegaly (CAPA) is a non-profit organization established spontaneously by patients with acromegaly and their families from all over the nation in 2012. Anyone who has been diagnosed and treated with acromegaly in China during the past three decades is welcome to join the CAPA. Therefore, as a representative national organization, the CAPA provides an ideal platform for large-scale surveys of Chinese patients.

Herein, we conducted the first nationwide comprehensive survey of 473 Chinese patients with acromegaly who were diagnosed and treated from 1996 to 2019 via the CAPA online platform. The objectives of this study were to describe in detail the demographics, clinical characteristics, diagnostic delays, treatment patterns, treatment outcomes, comorbidities, and treatment costs of patients with acromegaly in China.

## Materials and Methods

### Patients

This was a cross-sectional study. Patients with acromegaly aged 18 years or older at the time of diagnosis were included. All of the patients had been diagnosed with and treated for GH-secreting pituitary adenoma and were undergoing routine follow-up at hospitals.

In China, the biochemical diagnostic criteria for acromegaly were in accordance with the Endocrine Society clinical practice guidelines (12), namely, elevated IGF-1 levels and inability to suppress GH to 1 ng/ml following an oral glucose load of 75 g. In China, the first-line treatment for newly diagnosed patients is surgery, either via the most commonly adopted transsphenoidal approach or transcranial approach. For patients with surgical contraindications or an unwillingness to undergo surgery, medical treatment with a somatostatin analogue (SSA) or dopamine agonist (DA) served as alternative first-line treatment. After treatment, patients were required to complete routine follow-up, and hormone levels and magnetic resonance imaging (MRI) scans were re-evaluated. Patients with recurrent or residual tumors that were evaluated as resectable by MRI were recommended to undergo repeat surgeries, while those with unresectable tumors were recommended for medical treatment or radiotherapy. In the early stage after radiotherapy, when the hormone-lowering effect had not started, medical treatment could be temporarily added. These principles of treatment for acromegaly in China are consistent with international guidelines (12, 25). In this survey, biochemical control at follow-up was identified according to the consensus on cured acromegaly (26).

All data used in this analysis came from the survey of acromegaly in China performed at the end of 2019 and early 2020. China has 34 provincial administrative regions (PARs), including 23 provinces, 5 autonomous regions, 4 municipalities directly under the central government, and 2 special administrative regions (http://www.gov.cn/guoqing/2005-09/13/content_5043917.htm). Survey questionnaires were officially distributed to 600 patients across the nation on December 17, 2019 via the CAPA network platform, and 474 valid responses, including 442 questionnaires completed by patients themselves and 32 filled by the patients’ families, from 32 PARs were received by January 6, 2020. One respondent with an age of 16 years at diagnosis was excluded, and the survey data of the other 473 patients were finally included.

### Content and Design of the Survey

The survey questionnaire for acromegaly focused on 5 aspects: 1) demographics and clinical characteristics, 2) diagnostic delays, 3) treatment patterns and outcomes, 4) comorbidities and health status at follow-up, and 5) treatment costs. The demographics included sex, age, body mass index (BMI), highest degree of education, marital status, ethnicity, and place of residence. The clinical characteristics part investigated acromegaly-associated symptoms at diagnosis, disease duration from the onset of symptoms to diagnosis, and levels of GH and IGF-1 at diagnosis. The diagnostic delay part included the year of diagnosis, year of the first medical consultation due to acromegaly, whether there was a diagnostic delay, duration of the diagnostic delay, and the departments and hospitals that made the correct diagnosis of acromegaly. The patients’ perspectives on the difficulty of getting diagnosed with acromegaly were also rated in this part. In the treatment patterns part, we collected data on the treatments for acromegaly from the year of diagnosis to December 2019 among all patients, including microscopic transsphenoidal surgery, endoscopic transsphenoidal surgery, transcranial surgery, radiotherapy, and medical treatment with octreotide-long acting release (octreotide-LAR), lanreotide-autogel, pasireotide-LAR, other older types of somatostatin analogs, cabergoline, or bromocriptine. The questionnaire also collected information on the follow-up interval, levels of GH and IGF-1 at the last follow-up, and the latest symptoms, comorbidities, and health status. Since the specific AcroQoL questionnaire and the generic SF-12/SF-16 questionnaire were not able to acquire an objective and visualized parameter reflecting the overall QoL of patients, we adopted EuroQoL questionnaire visual analogue scale to evaluate the health status. The treatment costs part inquired about the total costs and out-of-pocket costs of surgery, medical treatment, and radiotherapy during 2018, as well as the patients’ medical insurance and personal and family annual income. Currency was initially provided as Chinese yuan (¥) in the questionnaires and was then converted to US dollars ($) in the analysis.

This survey was part of the 2019 China Rare Disease Survey, led by the China Alliance of Rare Diseases, implemented by the JC School of Public Health and Primary Care of the Chinese University of Hong Kong, co-sponsored by Peking Union Medical College Hospital, and coordinated by the Illness Challenge Foundation. The process and implementation and quality control of the survey were designed, monitored, and reviewed by the survey committee composed of senior medical specialists on acromegaly, leaders of the CAPA and our research team. The final survey questionnaire had undergone three rounds of pre-investigation with more than 10 respondents before its official release.

### Statistical Analysis

SPSS version 26.0 (IBM, USA) and Prism 8.4.3 (GraphPad Software, USA) were used to analyse the data and generate graphs. Categorical variables are shown as numbers and proportions. Comparisons of categorical variables were performed using the chi-squared test. Continuous variables are presented as the mean ± standard deviation or median plus interquartile range, according to the data distribution tested by Levene’s test. A nonpaired t-test was used to assess the differences between normally distributed continuous variables, and the Mann-Whitney U test was used for variables that failed the normality test. The false discovery rate (FDR) algorithm was used in multiple comparisons to control the chance of generating unwanted false positives. Statistical significance was defined as p < 0.05.

### Ethics

The institutional review board of the Chinese University of Hong Kong and the Ethical Committee of Peking Union Medical College Hospital approved the study protocol and informed consent form (Reference number: SBRE-18-268 and SK-814). All the participants signed an electronic consent form before entering the study.

## Results

### Demographics and Clinical Characteristics of Patients With Acromegaly in China

Data from 473 Chinese patients with acromegaly, including 278 females (58.8%) and 195 males (41.2%), were included (**Table 1**). The mean age was 39.4±9.5 years at diagnosis, with the majority of patients aged 25–54 years (n = 427; 90.3%) at diagnosis. The median levels of GH and IGF-1 at diagnosis were 17.0 ng/ml and 660.0 ng/ml, respectively. The median (mean) duration of acromegaly was 3 years (5 years), and 40% of all patients had a disease duration of over 5 years. The patients were diagnosed from 1996 to 2019; 50.1% of them were diagnosed before 2015, and 49.9% were diagnosed in or after 2015. Patients diagnosed in 2015 or later had a younger age at diagnosis (38.3±10.0 vs. 40.4±8.8 years, p = 0.014) and shorter disease duration (4.5 vs. 5.5 years, mean, p = 0.026) than those diagnosed before 2015. Among all patients, 44.2% had a bachelor’s degree or above, 81.6% were married, 95.8% belonged to the Han nationality, and 67.9% lived in a city/town.

**Table 1:** Patient characteristics of acromegaly in China.

	Patients with acromegaly (N = 473)
Age, years	39.4 ± 9.5
Body mass index, kg/m^2^	26.7 ± 7.1
GH at diagnosis, ng/ml	17.0 (6.0, 41.0)
IGF-1 at diagnosis, ng/ml	660.0 (450.0, 887.0)
Disease duration, years	3.0 (1.0, 7.0)
Age categories, n (%)	
18–24	12 (2.5)
25–34	158 (33.4)
35–44	171 (36.2)
45–54	98 (20.7)
55–64	27 (5.7)
65 or older	7 (1.5)
Sex, n (%)	
Male	195 (41.2)
Female	278 (58.8)
Disease duration categories, n (%)	
<2 years	138 (29.2)
≥2 years and <5 years	147 (31.1)
≥5 years	188 (39.7)
Time of diagnosis, n (%)	
Before 2015	237 (50.1)
In or after 2015	236 (49.9)
Education degree, n (%)	
Before college/University	264 (55.8)
College/University or higher	209 (44.2)
Marriage status, n (%)	
Married	386 (81.6)
Unmarried or divorced	87 (18.4)
Ethnicity, n (%)	
Han nationality	453 (95.8)
Minority nationality	20 (4.2)
Region, n (%)	
Rural area	152 (32.1)
Town/city	321 (67.9)

Continuous variables are presented as means ± S.D.s or medians (25^th^ and 75^th^ interquartile).

GH, growth hormone; IGF-1, insulin-like growth factor 1.

**Figure 1:**
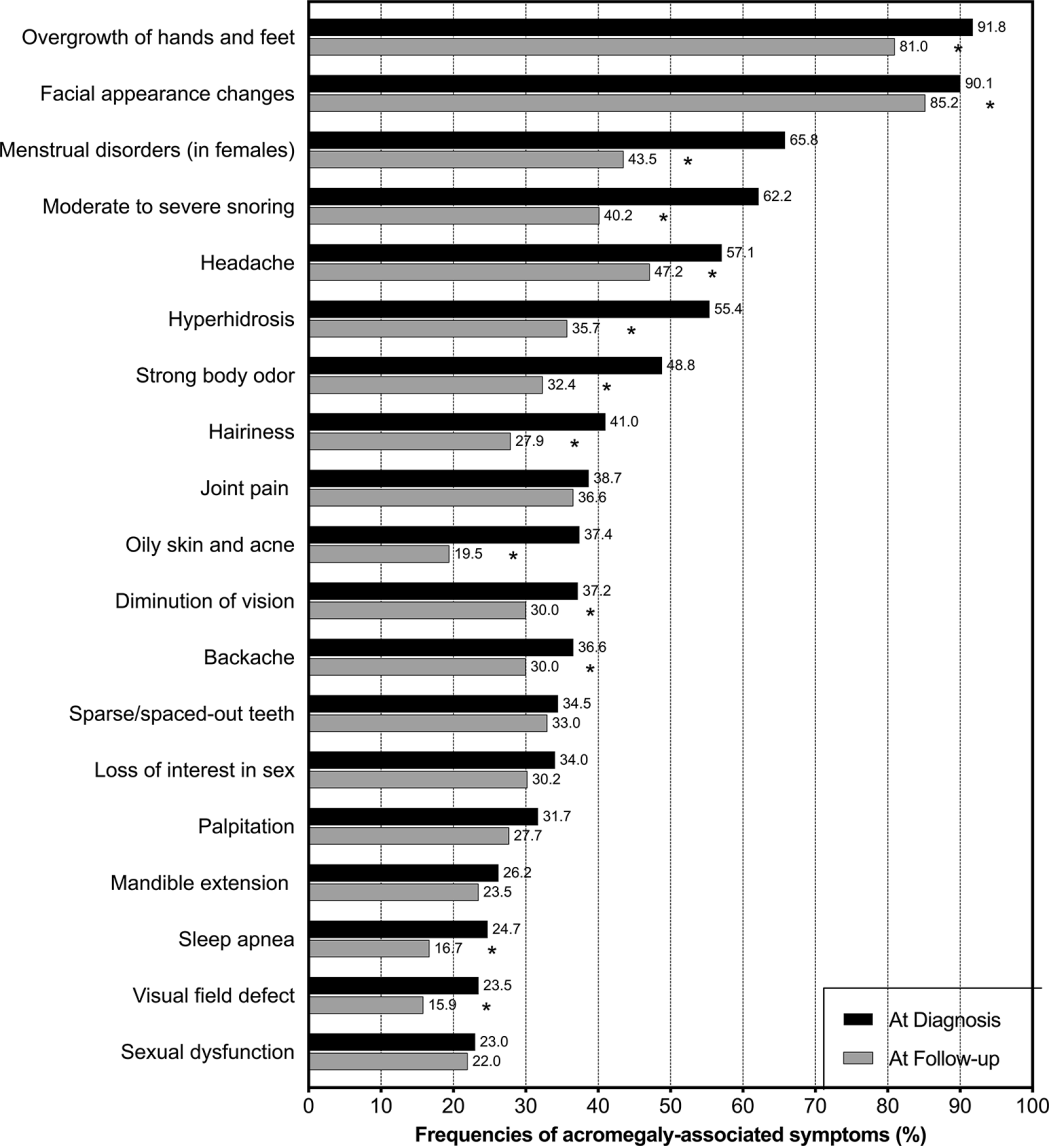
Frequencies of symptoms among patients with acromegaly in China. Black bars represent the frequencies of symptoms at the time of diagnosis, and grey bars represent the frequencies of symptoms at a median follow-up time of 5 years after treatment. * indicates that the reduction in frequency after treatment is significant (p < 0.05).

At least one symptom was present in 465 patients (98.3%) at the time of diagnosis. The median number of symptoms was 8. The symptoms that occurred in more than 20% of patients are illustrated in **Figure 1**. Among all patients, the most common symptoms at diagnosis in descending order were overgrowth of hands and feet (92%), facial appearance changes (90%), moderate to severe snoring (62%), headache (57%), and hyperhidrosis (55%). In female patients, the third most common symptom was menstrual disorders (66%). Of all patients, 96.6% had either facial appearance changes or overgrowth of the hands and feet.

### Diagnostic Delay Within the Healthcare System

The majority of patients (77.6%) received a diagnosis of acromegaly from departments of neurosurgery (48.0%) and endocrinology (29.6%). The other departments that diagnosed these patients included neurology (4.7%), otolaryngology (1.7%), oncology (1.7%), ophthalmology (1.5%), and gynecology (1.5%). Most patients (83.7%) were diagnosed in 3-A hospitals, the top level in China. The majority of patients (298, 63.0%) considered getting diagnosed with acromegaly “difficult”, with 45.3% of them rating the experience as “extremely difficult”.

Among all patients, 175 (37.0%) were clinically diagnosed with acromegaly at their first medical consultation. However, diagnostic delays occurred in 298 patients who received no suspected diagnosis of acromegaly at the first consultation (63.0%). Of these patients, 125 (41.9%) were treated as other diseases, and 67 (22.5%) were told there was no problem; referrals were only recommended for approximately one third of these patients (35.6%). More patients had a diagnosis delay less than 1 year than those with a diagnosis delay ≥1 year (63.8 vs. 36.2%, p < 0.001). Among patients with a diagnostic delay, 10% had a delay of 5 years or more. Compared to those without a diagnostic delay, patients with a diagnostic delay were more likely to be female (64.1 vs. 49.7%, p = 0.002), had more symptoms at diagnosis (7 vs. 5, median, p < 0.001), and had higher frequencies of headache (63.8 vs. 45.7%, p < 0.001) and menstrual disorders (in females, 75.4 vs. 44.8%, p < 0.001) (**Table 2**).

**Table 2:** Diagnostic delay in acromegaly and clinical correlations.

	Patients without diagnostic delay (N = 175)	Patients with diagnostic delay (N = 298)	P values
Age, years	39.8 ± 10.1	39.1 ± 9.1	0.473
Female, n (%)	87 (49.7)	191 (64.1)	0.002*
Body mass index, kg/m^2^	26.9 ± 7.6	26.7 ± 6.8	0.757
Disease duration, years	3.0 (1.0, 7.0)	4.0 (2.0, 7.0)	0.326
GH at diagnosis, ng/ml	14.0 (5.5, 32.0)	22.0 (7.9, 50.0)	0.373
IGF-1 at diagnosis, ng/ml	631.5 (457.0, 868.3)	700.0 (450.0, 897.0)	0.917
Diagnosed in or after 2015, n (%)	90 (51.4)	146 (49.0)	0.609
Median number of symptoms, n	7 (5, 9)	9 (6, 12)	<0.001*
Frequency of overgrowth of hands and feet, n (%)	157 (89.7)	277 (93.0)	0.216
Frequency of facial appearance changes, n (%)	155 (88.6)	271 (90.0)	0.406
Frequency of menstrual disorders in women, n (%)	39 (44.8)	144 (75.4)	<0.001*
Frequency of moderate to severe snoring, n (%)	104 (59.4)	190 (63.8)	0.349
Frequency of headache, n (%)	80 (45.7)	190 (63.8)	<0.001*
Frequency of hyperhidrosis, n (%)	87 (49.7)	175 (58.7)	0.057
Degree of college/university, n (%)	78 (44.6)	131 (44.0)	0.897
Married, n (%)	136 (77.7)	250 (83.9)	0.094
Minority nationality, n (%)	10 (5.7)	10 (3.4)	0.218
Living in rural areas, n (%)	54 (30.9)	98 (32.9)	0.648
Diagnosed in Neurosurgery, n (%)	87 (49.7)	140 (47.0)	0.565
Diagnosed in Endocrinology, n (%)	52 (29.7)	88 (29.5)	0.966
Diagnosed in other departments, n (%)	36 (20.6)	70 (23.5)	0.462
Diagnosed in 3-A hospitals, n (%)	152 (86.9)	244 (81.9)	0.157
Diagnosed in other hospitals, n (%)	23 (13.1)	54 (18.1)	0.157

Continuous variables are presented as means ± S.D.s or medians (25^th^ and 75^th^ interquartile).

*indicates the differences between two groups are statistically significant after being adjusted by FDR algorithm.

GH, growth hormone; IGF-1, insulin-like growth factor 1.

### Treatment Patterns

Out of all 473 patients, 79.3% (n = 375) had undergone surgery for acromegaly, all as first-line therapy; of these patients, 38.7% were treated via the transsphenoidal endoscopic approach (TEA), 37.4% via the transsphenoidal microscopic approach (TMA), and 3.2% via the transcranial approach (TCA). The proportion of surgeries via TEA was higher among patients diagnosed in or after 2015 than patients diagnosed before 2015 (47.9 vs. 29.5%, p < 0.001), while the proportion of surgeries via TMA was lower among patients diagnosed in or after 2015 than patients diagnosed before 2015 (30.1 vs. 44.7%, p = 0.001) (**Table 3**).

**Table 3:** Treatment patterns and outcomes in Chinese patients with acromegaly.

	Total patients (N = 473)	Patients diagnosed before 2015 (N = 237)	Patients diagnosed in or after 2015 (N = 236)	P values
**Treatment patterns**				
Surgery				
As first-line therapy/adjuvant therapy, n	375/0	185/0	190/0	–
Transsphenoidal microscopic approach, n (%)	177 (37.4)	106 (44.7)	71 (30.1)	0.001*
Transsphenoidal endoscopic approach, n (%)	183 (38.7)	70 (29.5)	113 (47.9)	<0.001*
Transcranial approach, n (%)	15 (3.2)	9 (3.8)	6 (2.5)	0.436
Medical treatment				
As first-line therapy/adjuvant therapy, n	97/197	55/108	42/89	–
Somatostatin analogues				
Octreotide-long acting release, n (%)	156 (33.0)	78 (32.9)	78 (33.1)	0.974
Lanreotide-autogel, n (%)	28 (5.9)	17 (7.2)	11 (4.7)	0.247
Pasireotide-long acting release, n (%)	17 (3.6)	16 (6.8)	1 (0.4)	<0.001*
Octreotide acetate, n (%)	15 (3.2)	6 (2.5)	9 (3.9)	0.426
Dopamine agonists				
Cabergoline, n (%)	74 (15.6)	42 (17.7)	32 (13.6)	0.213
Bromocriptine, n (%)	36 (7.6)	22 (9.3)	14 (5.9)	0.169
Radiotherapy				
As first-line therapy/adjuvant therapy, n	1/151	1/110	0/41	–
Total, n (%)	152 (32.1)	111 (46.8)	41 (17.4)	<0.001*
**Treatment outcomes**				
Follow-up time, years (median, interquartile)	5.0 (2.0, 7.0)	7.0 (6.0, 10.0)	2.0 (2.0, 4.0)	<0.001*
Number of symptoms at follow-up, n (%)	6 (4, 9)	7 (4, 10)	5 (3, 9)	0.002*
Biochemically controlled/Cured rate, n (%)^#^	138/299 (46.2)	59/139 (42.4)	79/160 (49.4)	0.275

^#^A total of 299 patients (139 diagnosed before 2015, and 160 in or after 2015) with complete follow-up records of hormones were included in this row.

*indicates the differences between patients diagnosed in different period are statistically significant after being adjusted by FDR algorithm.

Medical treatment was performed in 62.2% (n = 294) of all patients, including in 20.5% (n = 97) as first-line therapy and 41.7% (n = 197) as adjuvant therapy. Among all patients, the most commonly used SSAs were octreotide-LAR (33.0%), lanreotide-autogel (5.9%), pasireotide-LAR (3.6%), and octreotide acetate (3.2%), and the most commonly used DAs were cabergoline (15.6%) and bromocriptine (7.6%). The proportion of medical treatment with pasireotide-LAR significantly decreased among patients diagnosed in or after 2015 compared with those before 2015 (0.4 vs. 6.8%, p < 0.001).

Radiotherapy was provided to 32.1% of all patients (n = 152), including for 1 patient as initial therapy and 151 (99.3%) as adjuvant therapy. The proportion of radiotherapy among patients diagnosed in or after 2015 was significantly lower than those diagnosed before 2015 (17.4 vs. 46.8%, p < 0.001).

### Treatment Outcomes at Follow-Up

The median (interquartile range) follow-up time among all patients from diagnosis to this survey was 5.0 (2.0, 7.0) years. The most common follow-up intervals were three to six months (40.4%) or one year (33.0%).

Among the 299 patients with complete hormone records at the last follow-up, 46.2% (n = 138) satisfied the biochemical control (cure) criteria of acromegaly. Patients with controlled acromegaly (controlled patients) were older (41.1±9.8 vs. 37.8±9.7 years, p = 0.004) and more likely to be married (87.7 vs. 72.7%, p = 0.001) than those with uncontrolled acromegaly (uncontrolled patients) (**Table 4**). In addition, a biochemically uncontrolled status was correlated with living in a rural area (p = 0.025) and treatment with octreotide-LAR (p = 0.016), cabergoline (p = 0.023) and radiotherapy (p = 0.008).

**Table 4:** Patients with biochemically controlled or uncontrolled acromegaly and correlation analysis.

	Patients with controlled acromegaly (N = 138)	Patients with uncontrolled acromegaly (N = 161)	P values
Age, years	41.1 ± 9.8	37.8 ± 9.7	0.004*
Female, n (%)	84 (60.9)	96 (59.6)	0.827
Body mass index, kg/m^2^	26.5 ± 6.2	26.3 ± 7.1	0.778
Disease duration, years	4.0 (1.0, 7.0)	3.0 (2.0, 7.0)	0.985
GH at diagnosis, ng/ml	16.6 (5.1, 36.5)	16.0 (6.3, 43.3)	0.287
IGF-1 at diagnosis, ng/ml	680.0 (498.0, 947,.0)	644.0 (458.5, 868.3)	0.309
Diagnosed in or after 2015, n (%)	79 (57.2)	81 (50.3)	0.231
Number of symptoms at diagnosis, n	8 (6, 11)	9 (6, 11)	0.438
Degree of college/university, n (%)	61 (44.2)	66 (41.0)	0.576
Married, n (%)	121 (87.7)	117 (72.7)	0.001*
Minority nationality, n (%)	4 (2.9)	8 (5.0)	0.363
Living in rural areas, n (%)	34 (24.6)	59 (36.6)	0.025
Diagnostic delay, n (%)	97 (70.3)	100 (62.1)	0.137
Diagnostic delay < 1 year, n (%)	60 (43.5)	66 (41.0)	0.664
Diagnostic delay ≥ 1 year, n (%)	37 (26.8)	34 (21.1)	0.249
Diagnosed in Neurosurgery, n (%)	64 (46.4)	87 (54.0)	0.187
Diagnosed in Endocrinology, n (%)	45 (32.6)	39 (24.2)	0.108
Diagnosed in other departments, n (%)	29 (21.0)	35 (21.7)	0.879
Diagnosed and treated in 3-A hospitals, n (%)	112 (81.2)	138 (85.7)	0.289
Diagnosed and treated in other hospitals, n (%)	26 (18.8)	23 (14.3)	0.289
Treated with surgeries, n (%)			
Transsphenoidal microscopic approach	49 (35.5)	59 (36.6)	0.838
Transsphenoidal endoscopic approach	61 (42.2)	69 (42.9)	0.815
Transcranial approach	1 (0.7)	8 (5.0)	0.072
Treated with medical treatment, n (%)			
Somatostatin analogues			
Octreotide-long acting release	43 (31.2)	72 (44.7)	0.016
Lanreotide-autogel	7 (5.1)	13 (8.1)	0.300
Pasireotide-long acting release	9 (6.5)	5 (3.1)	0.163
Octreotide acetate	4 (2.9)	10 (6.2)	0.176
Dopamine agonists			
Cabergoline	19 (13.8)	39 (24.2)	0.023
Bromocriptine	15 (10.9)	10 (6.2)	0.147
Treated with radiotherapy, n (%)	38 (27.5)	68 (42.2)	0.008
Follow-up time, years	4.0 (2.0, 6.8)	4.0 (2.0, 7.0)	0.867
Number of symptoms at follow-up, n (%)	5 (3, 8)	7 (5, 10)	<0.001*

In this table, only 299 patients with complete follow-up records of hormones were included for analysis.

Continuous variables are presented as means ± S.D.s or medians (25^th^ and 75^th^ interquartile).

*indicates the differences between two groups are statistically significant after being adjusted by FDR algorithm.

GH, growth hormone; IGF-1, insulin-like growth factor 1.

The number of symptoms among patients was significantly lower at follow-up than at diagnosis (6 vs. 8, median, p < 0.001). Patients diagnosed in 2015 or later had fewer symptoms at follow-up than those diagnosed before 2015 (5 vs. 7, median, p = 0.002). The frequency of each symptom decreased after treatment (**Figure 1**), with significant decreases in overgrowth of hands and feet (p < 0.001), facial appearance changes (p = 0.023), menstrual disorders in women (p < 0.001), moderate to severe snoring (p < 0.001), headache (p = 0.002), hyperhidrosis (p < 0.001), strong body odor (p < 0.001), hairiness (p < 0.001), oily skin and acne (p < 0.001), the diminution of vision (p = 0.019), backache (p = 0.032), sleep apnea (p = 0.002), and visual field defects (p = 0.003).

**Figure 2:**
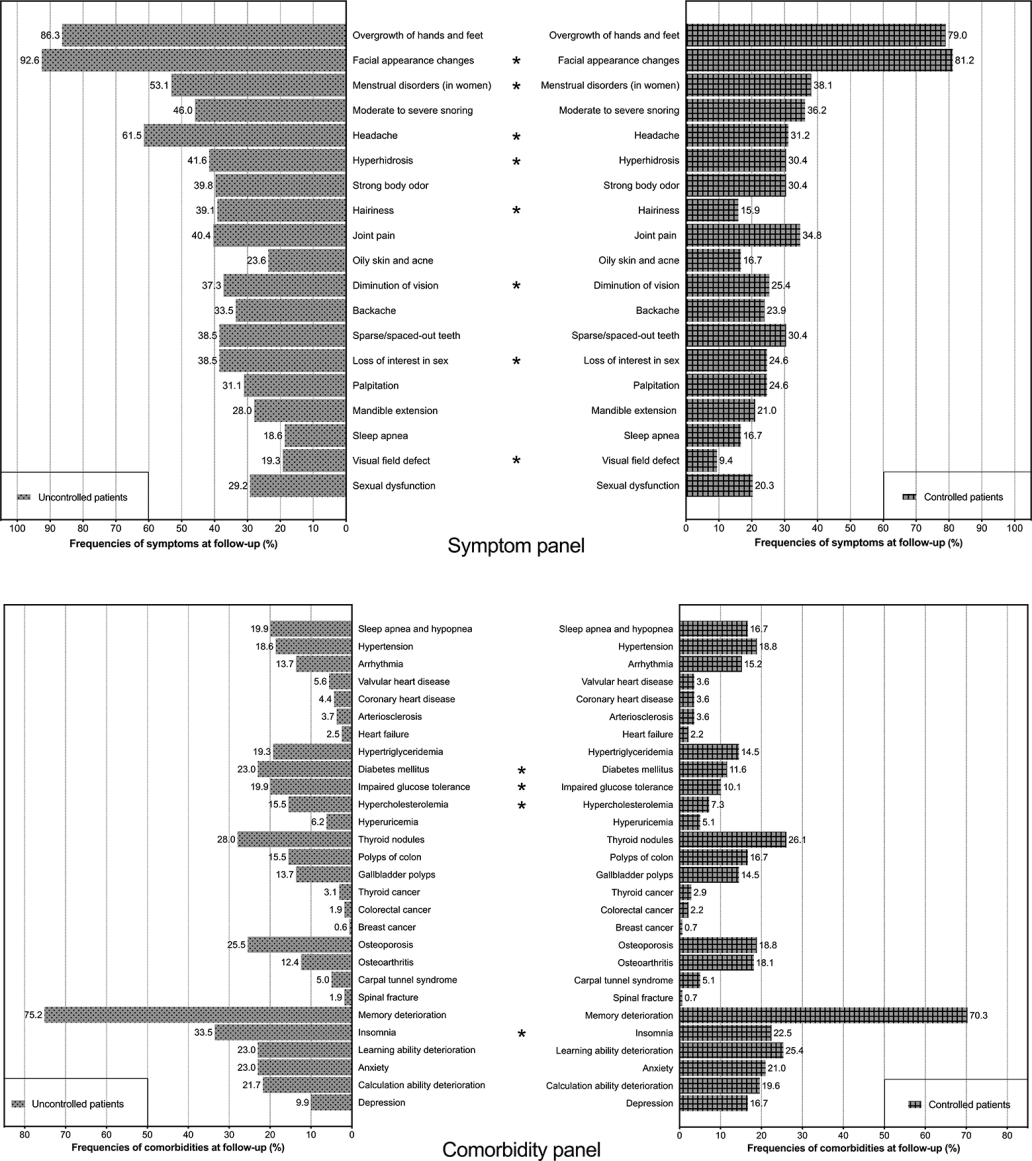
Frequencies of symptoms and comorbidities among controlled patients and uncontrolled patients at follow-up. The symptom panel (upper) includes two histograms showing the frequencies of symptoms among controlled patients and uncontrolled patients. The comorbidity panel (lower) includes two histograms showing the frequencies of comorbidities among controlled patients and uncontrolled patients. * indicates that the difference in frequency between controlled patients and uncontrolled patients is significant (p < 0.05).

Controlled patients had fewer symptoms than uncontrolled patients at follow-up (5 vs. 7, median, p < 0.001). Compared to uncontrolled patients (**Figure 2**, symptom panel), controlled patients had significantly lower frequencies of facial appearance changes (81.2 vs. 92.6%), menstrual disorders (in females) (38.1 vs. 53.1%), headache (31.2 vs. 61.5%), hyperhidrosis (30.4 vs. 41.6%), hairiness (15.9 vs. 39.1%), diminution of vision (25.4 vs. 37.3%), loss of interest in sex (24.6 vs. 38.5%), and visual field defects (9.4 vs. 19.3%) at follow-up.

### Comorbidities and Health Status at Follow-Up

Of all patients, 88.2% (n = 417) had at least one comorbidity at follow-up. The median number of comorbidities was 3. The twenty-eight comorbidities recorded among patients are listed in **Table 5**. The most common comorbidities were memory deterioration (71.2%), thyroid nodules (26.0%), insomnia (23.3%), reduced learning ability (21.6%), osteoporosis (21.4%), and anxiety (21.1%). Compared with uncontrolled patients (**Figure 2**, comorbidity panel), controlled patients had significantly lower frequencies of diabetes mellitus (11.6 vs. 23.0%), impaired glucose tolerance (10.1 vs. 19.9%), hypercholesterolemia (7.3 vs. 15.5%), and insomnia (22.5 vs. 33.5%).

**Table 5:** Acromegaly-associated comorbidities at follow-up.

	Patients with acromegaly (N = 473)
Cardiovascular and respiratory disease, n (%)	
Sleep apnea and hypopnea	76 (16.1)
Hypertension	73 (15.4)
Arrhythmia	64 (13.5)
Valvular heart disease	20 (4.2)
Coronary heart disease	17 (3.6)
Arteriosclerosis	14 (3.0)
Heart failure	10 (2.1)
Metabolic disorders, n (%)	
Hypertriglyceridemia	80 (16.9)
Diabetes mellitus	79 (16.7)
Impaired glucose tolerance	66 (14.0)
Hypercholesterolemia	58 (12.3)
Hyperuricemia	31 (6.6)
Neoplasms, n (%)	
Thyroid nodules	123 (26.0)
Polyps of colon	66 (14.0)
Gallbladder polyps	59 (12.5)
Thyroid cancer	11 (2.3)
Colorectal cancer	6 (1.3)
Breast cancer (in women)	2 (0.7)
Bone complications, n (%)	
Osteoporosis	101 (21.4)
Osteoarthritis	64 (13.5)
Carpal tunnel syndrome	22 (4.7)
Spinal fracture	7 (1.5)
Psychological and cognitive symptoms, n (%)	
Memory deterioration	337 (71.2)
Insomnia	110 (23.3)
Learning ability deterioration	102 (21.6)
Anxiety	100 (21.1)
Calculation ability deterioration	83 (17.5)
Depression	58 (12.3)

All patients were further divided into two groups according to the median number of comorbidities (**Table 6**). Compared to patients with <3 comorbidities, patients with 3 or more comorbidities were older (41.4±9.7 vs. 37.3±8.8 years), were more likely to be female (66.9 vs. 50.4%), had a higher BMI (27.6±8.4 vs. 25.9±5.3 kg/m^2^), were less likely to have a bachelor’s degree (36.8 vs. 51.7%), had a longer disease duration (4.0 vs. 3.0, median, years), and had more symptoms at diagnosis (9 vs. 7, median) and follow-up (5 vs. 4, median). Moreover, compared to patients with <3 comorbidities, diagnostic delays (73.2 vs. 52.6%), especially those of over a year (29.3 vs. 16.2%), were more common among patients with ≥3 comorbidities, and the proportion of patients diagnosed before 2015 was higher among patients with ≥3 comorbidities (56.5 vs. 43.6%, p = 0.005). Additionally, patients with ≥3 comorbidities were more likely to receive radiotherapy (38.9 vs. 25.2%) and had a longer follow-up period (5.0 vs. 4.0, median, years).

**Table 6:** Clinical relevance of comorbidities in acromegaly at follow-up.

	Patients with < 3 comorbidities (N = 234)	Patients with ≥ 3 comorbidities (N = 239)	P values
Age, years	37.3 ± 8.8	41.4 ± 9.7	<0.001*
Female, n (%)	118 (50.4)	160 (66.9)	<0.001*
Body mass index, kg/m^2^	25.9 ± 5.3	27.6 ± 8.4	0.011*
Disease duration, years	3.0 (1.0, 6.8)	4.0 (2.0, 8.0)	0.014*
GH at diagnosis, ng/ml	16.0 (6.2, 40.0)	19.0 (6.0, 45.0)	0.331
IGF-1 at diagnosis, ng/ml	716.0 (484.0, 893.0)	632.0 (409.5, 860.8)	0.330
Diagnosed before 2015, n (%)	102 (43.6)	135 (56.5)	0.005*
Number of symptoms at diagnosis, n	7 (5, 10)	9 (7, 12)	<0.001*
Degree of college/university, n (%)	121 (51.7)	88 (36.8)	0.001*
Married, n (%)	182 (77.8)	204 (85.4)	0.033
Minority nationality, n (%)	8 (3.4)	12 (5.0)	0.387
Living in rural areas, n (%)	81 (34.6)	71 (29.7)	0.253
Diagnostic delay, n (%)	123 (52.6)	175 (73.2)	<0.001*
Diagnostic delay < 1 year, n (%)	85 (35.0)	105 (43.9)	0.126
Diagnostic delay ≥ 1 year, n (%)	38 (16.2)	70 (29.3)	0.001*
Diagnosed in Neurosurgery, n (%)	119 (50.9)	108 (45.2)	0.217
Diagnosed in Endocrinology, n (%)	66 (28.2)	74 (31.0)	0.511
Diagnosed in other departments, n (%)	49 (20.9)	57 (23.8)	0.448
Diagnosed and treated in 3-A hospitals, n (%)	199 (85.0)	197 (82.4)	0.441
Diagnosed and treated in other hospitals, n (%)	35 (15.0)	42 (17.6)	0.441
Treated with surgeries, n (%)			
Transsphenoidal microscopic approach	90 (38.5)	87 (36.4)	0.643
Transsphenoidal endoscopic approach	93 (39.7)	90 (37.7)	0.641
Transcranial approach	5 (2.1)	10 (4.2)	0.204
Treated with medical treatments, n (%)			
Somatostatin analogues			
Octreotide-long acting release	66 (28.2)	90 (37.7)	0.029
Lanreotide-autogel	11 (4.7)	17 (7.1)	0.266
Pasireotide-long acting release	7 (3.0)	10 (4.2)	0.486
Octreotide acetate	5 (2.1)	10 (4.2)	0.204
Dopamine agonists			
Cabergoline	28 (12.0)	46 (19.2)	0.029
Bromocriptine	15 (6.4)	21 (8.8)	0.330
Treated with radiotherapy, n (%)	59 (25.2)	93 (38.9)	0.001*
Follow-up time, years	4.0 (2.0, 6.0)	5.0 (3.0, 8.0)	<0.001*
Number of symptoms at follow-up, n (%)	5 (3, 7)	8 (5, 11)	<0.001*

Continuous variables are presented as means ± S.D.s or medians (25^th^ and 75^th^ interquartile).

*indicates the differences between two groups are statistically significant after being adjusted by FDR algorithm.

GH, growth hormone; IGF-1, insulin-like growth factor 1.

Unemployment due to acromegaly occurred in 127 patients (26.8%). Patients with 3 or more comorbidities had higher unemployment rates than those with fewer comorbidities (32.6 vs. 20.9%, p = 0.004). The mean score of the health-related EuroQoL visual analogue scale, ranging from 0 (worse) to 100 (better), was 64.2±20.6. Patients with <3 comorbidities scored higher than those with more comorbidities (68.3±20.4 vs. 60.1±20.0, p < 0.001). Of all patients, 2.3% (n = 11) could not take care of themselves and thus needed help from family members; 4.4% (n = 21) used assistive devices, including joint protectors, bath stools, and non-invasive ventilators, frequently in their daily lives.

### Treatment Costs for Acromegaly and Income

A total of 303 patients were treated for acromegaly during 2018, including 112 with surgery, 259 with medical treatment, and 28 with radiotherapy. The per-capita cost of treatment in 2018 for these patients was $11013. Medical treatment, surgery and radiotherapy accounted for 67%, 29%, and 4% of the total treatment cost ($3336964), respectively. Medical insurance covered $1575358 (47%) in 2018, and the per-capita treatments cost after reimbursement was $5814.

The treatment-specific costs are summarized in **Table 7**. The personal out-of-pocket cost of radiotherapy ($2246, median) for patients treated with radiotherapy during 2018 was significantly lower than that of medical treatment ($3738, p = 0.027) for patients receiving medical treatment and that of surgery ($3658, p = 0.013) for patients who underwent surgery. Among patients who received medical treatment, uncontrolled patients had a higher total personal cost than controlled patients ($10140 vs. $6162, p = 0.005). A total of 73.7%, 86.6% and 85.7% of patients treated in 2018 had insurance for medical treatment, surgery, and radiotherapy, respectively. Among these patients, the median out-of-pocket cost of radiotherapy ($1811) was lower than those of medical treatment ($4202, p = 0.004) and surgery ($3477, p = 0.019).

**Table 7:** Treatment costs on acromegaly during 2018 in China.

	Medical treatment	Surgery	Radiotherapy
All patients recorded, n	259	112	28
Total personal cost, $	8040 (3035, 11590)	7243 (5312, 9656)	4092 (2898, 5907)
Out-of-pocket cost, $	3738 (1738, 5795)	3658 (2335, 5795)	2246 (1003, 4346)
Median (interquartile) self-paid rate, %	53.3 (39.2, 100.0)	50.0 (38.5, 68.9)	50.0 (37.4, 75.0)
Patients with medical insurance, n (%)	191 (73.7)	97 (86.6)	24 (85.7)
Total personal cost, $	10083 (5875, 11712)	7244 (5216, 9822)	4057 (2898, 5907)
Out-of-pocket cost, $	4202 (2608, 5795)	3477 (2028, 5085)	1811 (952, 3999)
Median (interquartile) self-paid rate, %	49.2 (33.1, 61.3)	46.3 (33.8, 60.6)	49.5 (37.2, 64.5)

The exchange rate between US dollars ($) and RMB (¥) referred to the rate announced on July 24, 2020.

Costs are presented as medians (25^th^ and 75^th^ interquartile).

The median personal and family annual incomes of all patients with acromegaly were $7248 and $14496, respectively. The personal annual income of patients with <3 comorbidities was significantly higher than that of patients with more comorbidities ($10873 vs. $6958, median, p = 0.025). Of the patients treated in 2018, 73.9% paid for medical expenses by themselves, 10.9% needed family help, and 15.2% borrowed money from relatives.

## Discussion

This is to the best of our knowledge the first nationwide comprehensive survey conducted in China focusing on the clinical features, diagnosis, treatment, outcomes, and economic burdens of acromegaly. Based on the data of 473 patients from 32 PARs across the country, this study provides a representative and detailed understanding of acromegaly in China from multiple angles and may serve as an initial step for developing and modifying acromegaly-related medical policies and for performing further longitudinal studies on health-related outcomes in China.

Acromegaly affects both males and females; many studies have demonstrated that females are more frequently affected, but some have indicated that both sexes are equally affected (9, 14, 15, 17, 18, 21, 22, 27–29). In the present study, females accounted for 58.8% of all patients, significantly larger than the percentage of males. The female sex was correlated with a diagnostic delay and more comorbidities at follow-up, consistent with previous results (8, 19, 27, 29). The average age of patients was 39 years, approximately 10 years younger than the patients from Sweden, France, Denmark and the United States but close to those from Korea and Bulgaria (14, 15, 23, 30–32). We showed that older age was suggested to be correlated with more comorbidities, in accordance with published literature (33, 34).

Patients with acromegaly exhibit a wide range of clinical symptoms due to the chronic influence of GH over-secretion and IGF-1 overproduction and tumor mass effects (4, 7, 27). Among the patients in our study, although 1.7% were asymptomatic, the vast majority had at least one symptom at diagnosis. Facial appearance changes and overgrowth of hands and feet were the two most common acromegaly-associated symptoms, both of which were detected in more than 90% of patients. Menstrual disorders were seen in two-thirds of female patients and were thus the third most common symptom in females. The other common symptoms included moderate to severe snoring, headache, hyperhidrosis, strong body odor, hairiness, joint pain, oily skin and acne, vision diminution, backache, sparse teeth, loss of interest in sex, etc., consistent with published large-scale studies (35–37).

Symptoms of acromegaly always overlap those of other diseases (27). Therefore, patients seek consultations in different departments (38). In this study, nearly half of the patients received a diagnosis of acromegaly at a department focusing on the pituitary subspecialty of neurosurgery, and 30% were diagnosed at the endocrinology outpatient service. Other patients were diagnosed during consultations for acromegaly-associated symptoms at other departments, i.e. headache at the neurology department, vision loss at the ophthalmology department, menstrual disorders at the gynecology department, and snoring at the otolaryngology department. Due to the popularization of biochemical measurements for GH and IGF-1 and easier online access to information on acromegaly, the disease duration has been reduced, as previously reported (2, 39, 40). The median disease duration among patients in this study was 3 years, which is shorter than the 4.5–5 years in other studies (2, 28, 35, 36, 38, 41). The diagnosis of acromegaly was not always made at the first medical consultation and was thus defined in this study as a diagnostic delay within the healthcare system. Several studies have indicated that early diagnosis occurs when medical specialists have adequate awareness of acromegaly (42). From this perspective, retraining healthcare providers who may encounter patients with acromegaly at office visits or strengthening patient-expert education in medical schools (38) might help reduce diagnostic delays within the healthcare system.

Surgical resection of pituitary adenomas, mostly through the transsphenoidal approach, is the recommended primary therapy for acromegaly (12). Microscopic transsphenoidal surgery has been the main approach for decades. However, since endoscopic surgery provides wider intraoperative views and leads to similar remission and complication rates compared to microscopic surgery, it has increasingly been adopted as an alternative or even preferred method worldwide (43). In the current study, the endoscopic-to-microscopic ratio transitioned from 29.5:44.7 before 2015 to 47.9:30.1 within the past 5 years in China, indicating the wide acceptance of endoscopic microsurgery among neurosurgeons. Pasireotide-LAR usage was remarkably reduced among patients diagnosed in the past 5 years. This decline could have been influenced by the shorter follow-up time in these patients but could also have been influenced by the updated experience that pasireotide-LAR should be used in patients with no response to traditional SSA treatment (44). The proportion of radiotherapy has also greatly reduced in the past 5 years. One possible reason is the update to treatment guidelines for acromegaly published in 2014 (12).

Several studies have investigated the predictors for acromegaly treatment outcomes, e.g., surgeon experience, patient age, granulation pattern, and adenoma characteristics on MRI (45–48). As shown in this study, an older age at diagnosis was correlated with biochemical control, consistent with the literature (47). Another factor that might be associated with an increased cure rate was living in towns/cities. Considering the national circumstances of China, that patients living in towns/cities had easier access to excellent pituitary tumor centers (49) and better financial support for treatment than those living in rural areas might be a reasonable explanation for this finding. Moreover, our results showed that controlled patients had a higher marriage rate than uncontrolled patients. We believed that on the one hand, spouses and kids would give extra support to married patients to help them complete treatments, and on the other hand, cured patients were more likely to maintain a healthy marriage.

In our study, the median number of symptoms among patients dropped from eight at diagnosis to six at follow-up, and the frequency of 13 out of 19 symptoms significantly decreased. Unlike the result that remission and symptom improvement are not associated with each other (50), we found that controlled patients had fewer symptoms after treatment and significantly lower frequencies of eight symptoms than uncontrolled patients, highlighting the essential role of a cure in relieving acromegaly-associated symptoms.

Hypertension, sleep apnea, hypertriglyceridemia, diabetes mellitus, thyroid nodules, colon polyps, osteoporosis and osteoarthritis were among the most common comorbidities in this population, similar to those published in other cohorts (3, 5, 6, 23, 51). Several studies demonstrated that high levels of GH and IGF-1 impaired psychological and cognitive function (3, 52–54). The results of our study revealed that reduced learning ability, anxiety and insomnia existed in more than one-fifth of patients and memory deterioration existed in over 70% of the patients at follow-up. This result unveiled and proved the considerable psychological and cognitive burdens on patients with acromegaly even after treatment (55).

The clinical relevance of the number of comorbidities has not been elucidated. One recent study showed that more comorbidities were associated with lower AcroQoL scores (56). Our result was similar in that patients with more comorbidities had lower scores on the EuroQoL visual analogue scale.

Disfigurement, physical problems, and psychological disorders caused by acromegaly and the frequent need for treatment contribute to unemployment among patients with acromegaly (14, 32, 57). In this population, over one-fourth of the patients became unemployed after being diagnosed with acromegaly. The finding that comorbidities affected unemployment and income was unexpected. Patients with more comorbidities were more likely to be unemployed and had lower personal annual incomes than those with <3 comorbidities. Provision and improvement of appropriate social services for patients in need of assistive devices, personal care services, and psychological consultations are needed.

Treatments for acromegaly contribute to substantial economic burdens for patients (14, 15). In this study, the mean personal cost of treatment was $11013 (€9333) among Chinese patients, which was lower than the €12000 to €14000 estimated in Western countries (14, 32). The same conclusion was reached by our study and by previous studies, that drugs for medical treatments were the largest contributors (67%) to cost (14, 32, 58). Interestingly, although the total economic burden of medical treatment was more than twice that of surgery, the personal costs of drugs in patients undergoing medical treatment was similar to that of surgery in patients undergoing surgical treatment, indicating that the large number of patients receiving medical treatment led to the huge economic burden associated with treatment.

Although the gross domestic product in China ranks second in the world, the per capita disposable income was only $4092 in 2018 (http://www.stats.gov.cn/). In patients treated in 2018, the personal and family annual incomes were $7248 and $14496, respectively, which were higher than the national per capita level. Only 85% of patients were able to pay the after-reimbursement treatment cost by themselves or with help from family members. Advocating for universal medical insurance, increasing the reimbursement amount, and reducing the unemployment rate to increase income would help patients complete multimodal treatments and increase the cure rate in China.

The current study has limitations. First, the results of this study were all derived from the survey data of 473 patients from the CAPA. Although these respondents were representative, as they were diagnosed between 1996 and 2019 and from 32/34 China’s PARs, there are inevitable gaps between their characteristics and the real-world data. The China Pituitary Adenoma Specialist Council (CPASC, http://www.cpasc.cn/) was established in 2012 with 80 excellent pituitary tumor centers across the country and aimed to analyse the clinical outcomes and standardize the diagnosis and treatment procedures of patients with pituitary adenomas, including acromegaly, in China. However, no publicly available patient data have been released yet. As the former head of CPASC, our institution is urging the group to speed up the data collection process and strive to complete large-scale studies based on the Chinese population. Second, the overall response rate for our questionnaire survey was approximately 80%, leading to patient selection bias into this study. Third, we only recorded the costs of surgery, medical treatment and radiotherapy in the treatment cost portion of the survey. Adding comorbidity treatment costs, medical examination expenses, and indirect expenses such as transportation fees, missed earnings and hospitalization expenses into the analysis might lead to more valuable findings for policy-makers.

## Conclusions

This nationwide study provided the first comprehensive and robust description of patients with acromegaly in China, serving as an initial and essential step for further population-based studies. The current study revealed the similarities and differences between China and other nations in terms of acromegaly-associated clinical practice and healthcare systems, and we believe that these findings suggest implications for the management of patients with acromegaly and better allocations of healthcare resources.

## Data Availability Statement

The original contributions presented in the study are included in the article/supplementary materials. Further inquiries can be directed to the corresponding authors.

## Ethics Statement

The studies involving human participants were reviewed and approved by the Institutional Review Board of the Chinese University of Hong Kong and the Ethical Committee of Peking Union Medical College Hospital (Reference number: SBRE-18-268 and SK-814). The patients/participants provided their electronic informed consent to participate in this study.

## Author Contributions

XG, KW, SY, BX, SZ, and DD designed the study. KW, SY, HZ, and DD performed the survey and collected responses. BX, HZ, SZ, and DD monitored the entire process and the implementation and quality control of the survey. XG, LG, and ZW performed the statistical analysis and visualization of the data. XG wrote the manuscript. DD and BX revised the manuscript, and the whole team approved the final version of the manuscript. All authors contributed to the article and approved the submitted version.

## Funding

This work was supported by the National Key Research and Development Program of China (grant number 2016YFC0901500) and the Center for Rare Diseases Research, Chinese Academy of Medical Sciences, Beijing, China (grant number 2016ZX310174-4).

## Conflict of Interest

The authors declare that the research was conducted in the absence of any commercial or financial relationships that could be construed as a potential conflict of interest.

## Abbreviations

BMI, body mass index; CAPA, Chinese Association of Patients with Acromegaly; CPASC, China Pituitary Adenoma Specialist Council; DA, dopamine agonist; FDR, false discovery rate; GH, growth hormone; IGF-1, insulin-like growth factor 1; LAR, long-acting release; MRI, magnetic resonance imaging; PAR, provincial administrative region; QoL, quality of life; SSA, somatostatin analogue; TA, transcranial approach; TEA, transsphenoidal endoscopic approach; TMA, transsphenoidal microscopic approach.
